# Supplementary material for: Gamma-Aminobutyric Acid Accumulation Contributes to Citrus sinensis Response against ‘Candidatus Liberibacter Asiaticus’ via Modulation of Multiple Metabolic Pathways and Redox Status
Source: Plants (Basel). 2023 Nov 2;12(21):3753. doi: 10.3390/plants12213753 (PMC10650511; doi:10.3390/plants12213753)
Supplement: Supplementary file 1 [file plants-12-03753-s001.zip › plants-2651426-supplementary.pdf]

plants

Supplementary Materials

Gamma-aminobutyric acid accumulation contributes to *Citrus sinensis* response against '*Candidatus* Liberibacter asiaticus' via modulation of multiple metabolic pathways and redox status

Yasser Nehela [1,2] and Nabil Killiny [1]

[1] Department of Plant Pathology, Citrus Research and Education Center, University of Florida, 700 Experiment Station Rd., Lake Alfred, FL 33850; nabilkilliny@ufl.edu (NK), yasser.nehela@agr.tanta.edu.eg (YN)
[2] Department of Agricultural Botany, Faculty of Agriculture, Tanta University, Tanta 31527, Egypt.
* Correspondence: nabilkilliny@ufl.edu

Table S1. Primers used for gene expression analysis by real time RT-PCR in this study [a].

Gene	Abbriviation	Pathway	Accession ID		Primer (Forward and Reverse)
**γ-Aminobutyrate (GABA) permease (aka amino-acid permease BAT1-like)**	*CsgabP*	GABA shunt	XM_006468698.3	F	TTCATCCCCGGACCTTTCAA
				R	CGAGCACTGAAGATCCAAGC
**GABA transaminase 3**	*CsgabT*	GABA shunt	XM_006481305.2	F	CTCCTGAATGGGGGATAGGT
				R	TGCTGGGACTTGAGTTCCTT
**GABA dehydrogenase (aka succinate-semialdehyde dehydrogenase [SSADH])**	*CsgabD*	GABA shunt	XM_006493686.2	F	CAACGTACATGGCGTGTCTC
				R	CTGGCCCATCATTTTCTGTT
**Glutamate decarboxylase-like**	*CsGAD*	GABA shunt	NM_001288909.1	F	ATAGCCCGACTGTTCAATGC
				R	ATTTCTCCCAGCAGACCTGA
**Glutamate decarboxylase 5-like**	*CsGAD5*	GABA shunt	XM_006478039.2	F	CCTTGACGGGAGAATTTGAG
				R	GCCGTACTTGTGACCACTGA
**Glutamate dehydrogenase 1**	*CsGDH1*	GABA shunt	XM_006479045.2	F	CTTTGAGTGGGTGCAGAACA
				R	CTCAAGCTTCCCAACCTCTG
**Glutamate dehydrogenase 2**	*CsGDH2*	GABA shunt	XM_006477017.2	F	CTCACCAGCAGTTGTTACCG
				R	CCAAAACCCTGGATAGCAAA
**5-Oxoprolinase**	*CsOXP1*	GABA shunt	XM_006478626.2	F	ATCCCTCCAGGCTCATTTCT
				R	CACTTCCACCTCCGATTGTT
**Glutamate synthase [NADH], amyloplastic**	*CsGS*	GABA shunt	XM_006490447.2	F	GCTCAACCCATGCGTATCTT
				R	CGAACCAGAAGCTCAAGGAC
**Glutamate 5-kinase**	*CsG5K*	GABA shunt	XM_006483017.2	F	ACGGTAACCAGACAGCAACC
				R	CCAGGTCTCTTGCTCCAATC
**γ-Glutamyltranspeptidase 1-like**	*Csγ-GT1-like*	GABA shunt	XM_006464249.2	F	GTGGCTCCAACAAACACAGA
				R	GCATAAAGGATCCTCCACCA
**γ-Glutamyltranspeptidase 3**	*Csγ-GT3*	GABA shunt	XM_006475300.2	F	TGGAGTTCCCGGTGAGATAG
				R	CCATTTGGTGCAAACACTTG
**γ-Glutamyltranspeptidase 3-like**	*Csγ-GT3-like*	GABA shunt	XM_006475299.2	F	TGGAGGACTCTGTTCCAACC
				R	CAGGCTCTGAGCAAGCTTTT
**γ-Glutamylcyclotransferase-1**	*Csγ-GCT* -1	GABA shunt	XM_006478365.2	F	ACTACGAGAGGCTCCCCATT
				R	TCGATTGCCACGTACTCTTG
**γ-Glutamylcyclotransferase-2**	*Csγ-GCT* -2	GABA shunt	XM_006484538.2	F	CAGCTTTATCGATCGCTTCC
				R	GGATCAGGTTGTGGTTCGAG
**Glutamate-cysteine ligase, chloroplastic** [b]	*Csγ-GCL*	GABA shunt	XM_006476514.1	F	CGGAGGATGCTGTTGTTGTA
				R	CTCCCAATCGAACCTCTCAG
**Glutaminyl-peptide cyclotransferase** [c]	*CsQPCT*	GABA shunt	XM_006490569.2	F	GCGTTCTTCAGTTCGGAGAG
				R	CCCCAACCATCTTTCATCTG
**Pyrrolidone-carboxylate peptidase 1** [d]	*CsPCP1*	GABA shunt	XM_006480219.2	F	CCATTTTTCCTGCTGATGGT
				R	GGCACATGAACGAAGAGTGA
Tyrosine decarboxylase 1	*CsTDC1*	Amino acids	XM_006479363.2	F	GCTCAGTGCTGGTCTCAACA
				R	CCTTATCACGAGCAGCCAAT
**Arginine decarboxylase-like**	*CsADC*	Amino acids	XM_006487236.2	F	GTCCCTTCAGTCAGCCTTTG
				R	TGCACAAGCAACTCATAGCC
**Argininosuccinate lyase, chloroplastic**	*CsASL*	Amino acids	XM_006472396.2	F	TGAGCCAAAGCACAAGACTG
				R	GTCGCTGATCAAACCCTGTT
**Argininosuccinate synthase, chloroplastic**	*CsASS*	Amino acids	XM_006476944.2	F	AGTCTCGGGAATCCCTGTTT
				R	AAGGACTCAAGCTCCTGCAC
**δ-1-Pyrroline-5-carboxylate dehydrogenase 12A1**	*CsP5CDH*	Amino acids	XM_006476581.1	F	TGCCAAAGGTCTCCAATTTC
				R	GGACCATAAGGCCAACGATA
**Proline dehydrogenase 1, mitochondrial-like**	*CsProDH*	Amino acids	XM_006482264.1	F	TTGCAGGGATTTCTCCAAAC
				R	CTGCAATCCGAGAAAAGAGG
**Serine acetyltransferase 1, chloroplastic-like**	*CsSAT1*	Amino acids	XM_006474855.2	F	AAGAGACCCTGCGTGCATAA
				R	AACAGTAACCCACGTCCGAT
**Serine acetyltransferase 5**	*CsSAT5*	Amino acids	XM_006475346.2	F	GAGCTCAGGTACCCATCTCC
				R	AGAGAGTGGAGAGAAGCGTG
**Cysteine synthase**	*CsCysK*	Amino acids	XM_006471676.1	F	CCAGCTGCCTTGACATTGTT
				R	AGCAGGGTTCCATCATCTCC
**Methionine synthase 2** [e]	*CsMS2*	Amino acids	XM_006479085.2	F	GTCCATGGAGAGCCTGAGAG
				R	ATGGTGACAGGGCCAGTAAG
**Cystathionine gamma-synthase 1, chloroplastic**	*CsCGS*	Amino acids	XM_006482566.3	F	AGATGGAACCTTTGCCACAC
				R	GAGAGCACCACCCAAAACAT
**Cystathionine beta-lyase, chloroplastic**	*CsCBL*	Amino acids	XM_006492446.3	F	GCAGGTGTGCTTGCTGTAAA
				R	GTCCAGGATGTTCAGGGAGA
**Chorismate synthase**	*CsCS*	Salicylic acid	XM_006485798.2	F	ACTGGAACACCCATCCATGT
				R	GAAATGAAGCCATCCCAGAA
**Chorismate mutase**	*CsCM*	Salicylic acid	XM_006482655.2	F	GGGAGCTTGCTTGATTCTTG
				R	GTACGCAGCTTGATGTTGGA
**Arogenate dehydratase/prephenate dehydratase 1, chloroplastic**	*CsADT*	Salicylic acid	XM_006467360.2	F	TTGGTGCCTAACAGATGTGG
				R	CCTTGTGAAAGCCAGAGGAC
**Aspartate aminotransferase, cytoplasmic-like**	*CsAST*	Salicylic acid	XM_006476023.2	F	GCTTCTCCAAGCATCTGGTC
				R	GTTTGAGCGGACTCCATCAT
**Tyrosine aminotransferase**	*CsTAT*	Salicylic acid	XM_006469841.1	F	GTGGAATTTCAGGGCTATCG
				R	TCGAGATGGCTATGTGTTGC
**Isochorismate synthase**	*CsICS*	Salicylic acid	XM_006476586.2	F	TGCCGGAACAGGGATAGTAG
				R	CTTGGTGAACTGCACAATGG
**Phenylalanine ammonia-lyase**	*CsPAL*	Salicylic acid	XM_006481431.2	F	GTTCTGGCCTGGCTTCTATG
				R	ATAAGAGCTGCCATCGAGGA
**3-Ketoacyl-CoA thiolase 2**	*CsKAT2*	Salicylic acid	XM_006489736.1	F	GGGTACAGTTTTGGCACCAG
				R	CAGGCACAGTTTCTGAACCA

**Alcohol acyl transferase**	*CsAAT*	Salicylic acid	NM_001288910.1	F	CTCGTGTTGATGTGCGTCTT
				R	TGGCTCTCCGGAACTATTA
***ω*-3-Fatty acid desaturase**	*CsFAD*	Jasmonic acid	XM_006480990.2	F	CAGATCCCGCATTACCACTT
				R	GGTATCGGTCCCGATTTCTT
**Lipoxygenase**	*CsLOX*	Jasmonic acid	XM_006483993.1	F	TGGCTGTCCAAGACACTCTG
				R	CAGCACCACATCTGCCTTTA
**Allene oxide synthase**	*CsAOS*	Jasmonic acid	NM_001288906.1	F	GTTTCAGCTCGCTCCGTTAC
				R	GAGGTTGTGACACGCTTCCT
**Allene oxide cyclase**	*CsAOC*	Jasmonic acid	NW_006260521.1	F	GCGAGTGGGAATTACAGCAG
				R	TTAACCTGCCCACTCACTCC
**Acetate/butyrate--CoA ligase AAE7**	*CsAAE7*	Jasmonic acid	XM_006488806.2	F	CCACCAGAGGACACAATCCT
				R	CTGAGCTTCGAAAGGGAGTG
**12-Oxophytodienoate reductase 3**	*CsOPR3*	Jasmonic acid	XM_006475468.2	F	TGCCACTTCTCTTGTTGTGG
				R	AAATGGTAGCGTCCCTTCCT
**Acyl-coenzyme A1**	*CsACX1*	Jasmonic acid	XM_006477083.2	F	ACGGATCATTGAGCTTCGTC
				R	CCCAGTGGAGATCCGTAAAA
**Enoyl-CoA hydratase, mitochondrial-like**	*CsAIM*	Jasmonic acid	XM_006488772.2	F	TAATCCTCGTGACCCGAGAC
				R	CTCTGCTGACGTCAAATCCA
**3-Ketoacyl-CoA thiolase , peroxisomal-like**	*CsKAT*	Jasmonic acid	XM_006480138.2	F	ATACCTGCTGCGGTGAAGTC
				R	AGGATCAAGCCCCAGTTTCT
**Anthranilate synthase alpha subunit 1**	*CsASA*	Auxins	XM_015532734.1	F	TTGAGCTTCAACCGGAGACT
				R	ACTGAAAGTTGCTGCGGACT
**Anthranilate synthase beta subunit 2**	*CsASB*	Auxins	XM_006469235.2	F	CAATCGCACAGTTGCTCAGT
				R	GCTCAGGATTGCAGAGAGA
**Tryptophan synthase alpha chain, chloroplastic-like**	*CsTSA*	Auxins	XM_006470948.2	F	TACGGAAGGAAGCCATCAAG
				R	GGAGTGGTGGGACCTGTAAA
**Tryptophan synthase beta chain 1, chloroplastic-like**	*CsTSB*	Auxins	XM_006493882.2	F	GTGTTGCATGGTGCTTTGAG
				R	CAGGTCCAACTCCAGGGTAA
**Tryptophan aminotransferase-related protein 2-like**	*CsTAA2*	Auxins	XM_015529685.1	F	AATGGAACTGGGTTGTCTGG
				R	TCATCAGGGATATGGGAAGG
**Tryptophan aminotransferase-related protein 4-like**	*CsTAA4*	Auxins	XM_006473060.2	F	CGCATCTGGTTGAGTTTGTG
				R	TAGAGCCAGCATGACCAGTG
**Indole-3-pyruvate monooxygenase YUCCA2**	*CsYUC2*	Auxins	XM_006466708.2	F	CTGGCCCTGTAATTGTTGGT
				R	ACGAGGGAATGGCACATAAG
**Indole-3-pyruvate monooxygenase YUCCA8**	*CsYUC8*	Auxins	XM_006480095.2	F	CTACAGGGTACCGCAGCAAT
				R	ATTTCCTTTCCAGCCATGTG
**Aromatic-L-amino-acid decarboxylase-like (aka tryptophan decarboxylase)**	*CsTDC1*	Auxins	XM_006469752.3	F	CGACTTTATGGCCTGGAAAA
				R	GGGGTGACAACCTATCCTCA
**Indole-3-acetaldehyde oxidase-like (aka Acetaldehyde oxidase)**	*CsAO1*	Auxins	XM_006487737.2	F	GGGATCAGACAGGAATCGAA
				R	CCGCCCCAAGAAGTATTGTA
**Zeaxanthin epoxidase**	*CsZEP*	Abscisic acid	XM_006466537.2	F	GCCGTTGTGCTTCTAGGTTC
				R	CGATCACCTTAACCCGAAAA
**Violaxanthin de-epoxidase**	*CsVDE*	Abscisic acid	NM_001288881.1	F	AATCGCATACCAACCTGCTC
				R	GGTTTGAAGGCAAGCAACAT
**Neoxanthin synthase**	*CsNSY*	Abscisic acid	NM_001288932.1	F	AACGTTGACCAAACCAGACC
				R	CTCACTGAGCACCGAAAGTG
**9-*cis*-Epoxycarotenoid dioxygenase 3**	*CsNCED*	Abscisic acid	NM_001288935.1	F	ATGGCGGCAGCAACTACTAC
				R	CTGCAGGTGATGGAGGGTAT
**Short chain alcohol dehydrogenase**	*CsABA2*	Abscisic acid	NM_001288867.1	F	GCAATCTGCTGGGCAATAGT
				R	ATGCCAGTAGCTCCACCTGT
**Abscisic aldehyde oxidase**	*CsAAO3*	Abscisic acid	XM_006487736.2	F	TTCCTTCAGGGGATTCTGTG
				R	CAACCTGTTTGGGGTACCAG
**1-Aminocyclopropane-1-carboxylate (ACC) synthase-like**	*CsACS*	Ethylene	NM_001288902.1	F	CGAATCCAGGGTGGTTTAGA
				R	TCCTTCGAGATGACATGCTG
**ACC oxidase**	*CsACO*	Ethylene	NM_001288883.1	F	ACCTCGGTGACCAAATTGAG
				R	TTTTTCAAGCAATGCTGGTG
***S*-Adenosylmethionine decarboxylase**	*CsSAMDC*	Ethylene	NM_001288936.1	F	CCTGAAGGAAAGGGCCTTAG
				R	GGTGGGATTGAAAGAAGCAA
**L-ascorbate peroxidase, cytosolic isoform X1**	*CsAPX*	Antioxidant	XM_015533381.2	F	CATAGCGAGCTTGTGCTCTG
				R	ATGAGTGGAGCGCAGTTCTT
**phospholipid hydroperoxide glutathione peroxidase**	*CsGPX*	Antioxidant	XM_006476535.2	F	CAAGCATCTGAAATCCAGCA
				R	GCAAGAAGCTGATCATGCAA
**cationic peroxidase 1-like**	*CscPOX*	Antioxidant	XM_006481093.2	F	GTGGCCACACTATTGGCTTT
				R	AGTAGCCCTCGTTTCTGCAA
**lignin-forming anionic peroxidase-like**	*CsliPOX*	Antioxidant	XM_006470880.2	F	GCTACCAAAGGCTTGAATGC
				R	ACCATTAGCCGGACACTGAC
**peroxidase A2-like**	*CsPOX-A2*	Antioxidant	XM_006494741.3	F	GCAATCCAGACCCAACACTT
				R	TATTGACAATGGCAGCGGTA
**peroxidase 3**	*CsPOX3*	Antioxidant	XM_006469646.3	F	TTGAAATGGACCCTGGTAGC
				R	CAGACTTGGCAAATTCAGCA
**superoxide dismutase [Cu-Zn], chloroplastic**	*CsSOD-Cu/Zn*	Antioxidant	XM_006487937.3	F	GGCTCACGTTCTCCTCGTAG
				R	AGAGGGGAATTTGAGGGAGA
**superoxide dismutase [Mn], mitochondrial**	*CsSOD-Mn*	Antioxidant	XM_006486115.2	F	GGGCTATTGACACGCATTTT
				R	ACCAGTGGATCCTGATTTGC
**superoxide dismutase [Fe] 2, chloroplastic-like**	*CsSOD-Fe*	Antioxidant	XM_006484979.3	F	CAGTGCCCATTACACATTGC
				R	TCTCCATGGAAATTGGAAGC
**superoxide dismutase [Fe] 3, chloroplastic isoform X1**	*CsSOD-Fe3*	Antioxidant	XM_006484439.3	F	GGAATGACATCCCCATCATC
				R	ACATCCTTTGCTTGCAGCTT
**catalase-like isoform X1**	*CsCAT*	Antioxidant	XM_006473726.2	F	AAGGCTTGCATTGAGAAGGA
				R	CGGCCTGAGACCAGTAAGAG

**Elongation factor-1 alpha**	*CsEF1*	Reference gene	AY498567.1	F	GGAAGTTCGAGACCACCAAG
				R	ACACCAAGGGTGAAAGCAAG
**F-Box/kelch-repeat protein**	*CsF-box*	Reference gene	XM_006482390.1	F	ACTTGACAGATGGGCTGTCC
				R	CAGCAACCAAATACCCGTCT

[a] The listed genes were assembled based on recent available data in GenBank, National Center for Biotechnology Information website (NCBI, http://www.ncbi.nlm.nih.gov/gene/).

[b] γ-Glutamylcysteine synthetase (*γ-GCS*) from *Arabidopsis thaliana* has been matched as PREDICTED: *Citrus sinensis* glutamate-cysteine ligase, chloroplastic (*CsGCL*) using the protein-protein BLAST, based on recent available data in GenBank, National Center for Biotechnology Information website (NCBI, http://www.ncbi.nlm.nih.gov/gene/).

[c] Glutaminyl cyclase (*QC*) from *Arabidopsis thaliana* has been matched as PREDICTED: *Citrus sinensis* glutaminyl-peptide cyclotransferase (*CsQPCT*) using the protein-protein BLAST, based on recent available data in GenBank, National Center for Biotechnology Information website (NCBI, http://www.ncbi.nlm.nih.gov/gene/).

[d] Pyroglutamyl-peptidase (*PGP*) from *Arabidopsis thaliana* has been matched as PREDICTED: *Citrus sinensis* pyrrolidone-carboxylate peptidase 1 (*CsPCP1*) using the protein-protein BLAST, based on recent available data in GenBank, National Center for Biotechnology Information website (NCBI, http://www.ncbi.nlm.nih.gov/gene/).

[e] Methionine synthase 2 (*MS2*) from *Arabidopsis thaliana* has been matched as PREDICTED: Citrus sinensis 5-methyltetrahydropteroyltriglutamate-homocysteine methyltransferase 1 using the protein-protein BLAST, based on recent available data in GenBank National Center for Biotechnology Information website (NCBI, http://www.ncbi.nlm.nih.gov/gene/).

[f] Ornithine transcarbamylase (*OTC*) from *Arabidopsis thaliana* has been matched as PREDICTED: *Citrus sinensis* ornithine carbamoyltransferase, chloroplastic (*CsOTC*) using the protein-protein BLAST, based on recent available data in GenBank, National Center for Biotechnology Information website (NCBI, http://www.ncbi.nlm.nih.gov/gene/).

[g] Para-aminobenzoic acid synthase (*PABA synthase*) from *Arabidopsis thaliana* has been matched as PREDICTED: *Citrus sinensis* aminodeoxychorismate synthase, chloroplastic (*CsADCS*) using the protein-protein BLAST, based on recent available data in GenBank National Center for Biotechnology Information website (NCBI, http://www.ncbi.nlm.nih.gov/gene/).
